# Aerobic Physical Activity and a Low Glycemic Diet Reduce the AA/EPA Ratio in Red Blood Cell Membranes of Patients with NAFLD

**DOI:** 10.3390/nu10091299

**Published:** 2018-09-13

**Authors:** Valeria Tutino, Valentina De Nunzio, Maria Gabriella Caruso, Caterina Bonfiglio, Isabella Franco, Antonella Mirizzi, Giampiero De Leonardis, Raffaele Cozzolongo, Vito Giannuzzi, Gianluigi Giannelli, Maria Notarnicola, Alberto R. Osella

**Affiliations:** 1 Laboratory of Nutritional Biochemistry, National Institute of Gastroenterology “S. de Bellis” Research Hospital, 70013 Castellana Grotte (Ba), Italy; valeria.tutino@irccsdebellis.it (V.T.); valentinadx@hotmail.it (V.D.N.); 2 Ambulatory of Clinical Nutrition, National Institute of Gastroenterology “S. de Bellis” Research Hospital, 70013 Castellana Grotte (Ba), Italy; gabriella.caruso@irccsdebellis.it (M.G.C.); giampierodl@gmail.com (G.D.L.); 3 Laboratory of Epidemiology and Biostatistics, National Institute of Gastroenterology “S. de Bellis” Research Hospital, 70013 Castellana Grotte (Ba), Italy; catia.bonfiglio@irccsdebellis.it (C.B.); isabella.franco@irccsdebellis.it (I.F.); antonella.mirizzi@irccsdebellis.it (A.M.); arosella@irccsdebellis.it (A.R.O.); 4 Department of Gastroenterology 1, National Institute of Gastroenterology “S. de Bellis” Research Hospital, 70013 Castellana Grotte (Ba), Italy; raffaelecln1@alice.it (R.C.); v55567@alice.it (V.G.); 5 Scientific Direction, National Institute of Gastroenterology “S. de Bellis” Research Hospital, 70013 Castellana Grotte (Ba), Italy; gianluigi.giannelli@irccsdebellis.it

**Keywords:** physical activity, polyunsaturated fatty acids, erythrocyte membrane, non-alcoholic fatty liver disease

## Abstract

Omega-6 Polyunsaturated Fatty Acids (PUFAs), through the eicosanoids derived from arachidonic acid (AA), are able to modulate the inflammatory processes, whereas omega-3 PUFAs, such as eicosapentaenoic acid (EPA), exert anti-oxidant and anti-inflammatory effects. An unbalanced AA/EPA ratio in favor of AA leads to the development of different metabolic disorders, including non-alcoholic fatty liver disease (NAFLD). The aim of the present study was to evaluate the effects of different diets, alone and in combination with two physical activity programs, on the AA/EPA ratio value in erythrocyte membranes of patients with NAFLD. One hundred forty-two subjects with NAFLD were enrolled in the study and randomized into six treatment groups. AA/EPA ratio was significantly reduced after 90 days of treatment with only a program of aerobic activity. However, it appears that the combination of physical activity and a Low Glycemic Index Mediterranean Diet (LGIMD) was more efficacious in reducing AA/EPA levels, at 45 days of treatment, even if this effect was not maintained over time. The combined effect of diet and physical activity reduced the AA/EPA ratio value improving the score of steatosis. Dietary intake of omega-3 PUFAs, in association with a healthy lifestyle, may be used in the prevention protocols for many chronic diseases, including NAFLD.

## 1. Introduction

Omega-3 and omega-6 Polyunsaturated Fatty Acids (PUFAs) are a family of lipids identified by position of the first double bond with respect to the methyl end of the fatty acid molecule. Omega-3 PUFAs include alpha-linolenic acid (ALA), eicosapentaenoic acid (EPA), and docosahexaenoic acid (DHA), while linolenic acid (LA) and arachidonic acid (AA) are examples of omega-6 PUFAs. LA and ALA are considered essential fatty acids (EFA) because they cannot be synthesized by humans and thus must be obtained from diet [1,2]. Omega-3 fatty acids should be consumed in a balanced ratio of 1:1 or 2:1 with omega-6 fatty acids [1,2,3], however, it has been demonstrated that in typical Western diet the omega-6 and omega-3 PUFAs are consumed in a ratio of 15/20:1, and these high levels of omega-6 PUFAs can promote the development of many inflammatory diseases [3,4]. PUFAs are able to modulate the inflammatory processes: omega-6 fatty acids have a pro-inflammatory and pro-thrombotic action mediated by eicosanoids derived from AA, and this action can be suppressed by omega-3 PUFAs, such as EPA, that exert anti-oxidant effects [2,4]. Since AA and EPA represent the active biological forms of omega-6 and omega-3 PUFAs respectively, their ratio is considered a specific index to better evaluate the PUFAs status in both whole blood and red blood cells (RBC) membrane phospholipids [5,6,7]. In a study conducted on both healthy subjects and patients with different pathologies, such as allergic, neurodegenerative, skin, inflammatory, heart, metabolic and cancer diseases, the AA/EPA ratio was demonstrated to be more sensitive and accurate than the omega-6/omega-3 PUFAs [5]. The AA/EPA ratio is related to food intake and it is the most reliable indicator of nutritional status [2]. Several studies have shown that an unbalanced AA/EPA ratio in favor of AA fatty acid, leads to the development of different metabolic disorders, including obesity, cardiovascular disease (CVD) and non-alcoholic fatty liver disease (NAFLD) [8,9,10,11,12]. In NAFLD, a liver manifestation of Metabolic Syndrome (MetS) characterized by excessive hepatic fat deposition, changes in fatty acid composition of cell membranes have been observed [13,14]. In a previous study, we showed the presence of low levels of Saturation Index (SI), given by the relationship of stearic acid to oleic acid, in RBC membranes of patients with severe NAFLD compared to controls [14], suggesting that SI is an indicator of liver injury. Several studies report that omega-3 PUFAs are able to prevent the development of NAFLD by limiting the preservation of triacylglycerol in the liver, therefore a higher omega-3/omega-6 PUFAs ratio in the diet is essential for improving human health [9].

In the light of this experimental evidence, the present study was designed to probe the effects of two diets and two physical activity programs, as well as their combined action, on the AA/EPA ratio assessed in RBC membranes of patients with NAFLD.

## 2. Methods

### 2.1. Participants

One hundred forty-two subjects with NAFLD were recruited by the Laboratory of Epidemiology and Biostatistics of the National Institute of Digestive Diseases, IRCCS “S. de Bellis”, Castellana Grotte, Italy, from March 2015 to December 2016. The patients were enrolled consecutively in a nutritional trial (registration number CT02347696), that is part of the NUTRIATT (NUTRItion and Ac-TiviTy) study. Before participation, all subjects provided an informed consent form and the study was conducted in accordance with the Helsinki Declaration and approved by the Ethical Committee (Prot.n. 10/CE/De Bellis, 3 February 2015).

### 2.2. Study Design

This study was a parallel group randomized controlled clinical trial. Trial inclusion criteria were: Body Mass Index (BMI) ≥ 25, expressed as weight in kilograms divided by the square of height in meters (kg/m^2^); age > 30 years old and <60 years old; NAFLD moderate or severe. Subjects with stroke, clinical peripheral artery disease, evident cardiovascular diseases and subjected to revascularization procedures, were excluded from the study. Other exclusion criteria included: current treatment with insulin or oral hypoglycemic drugs; fasting glucose > 126 mg/dL, or casual glucose > 200 mg/dL; more than 20 g/daily of alcohol intake; severe medical condition that may impair the person to participate in a nutritional intervention study; people following a special diet or involved in a weight loss program, or who have suffered a recent weight loss, and those who cannot follow a diet for religious or other reasons.

### 2.3. Sample Size

Sample size was estimated considering the repeated measurement nature of the outcome. From a previous study [15], the mean ± Standard Deviation (SD) score of NAFLD was estimated to be 4.5 (1) and 4.0 (0.5) for the treatment and control group respectively; the type I error was fixed at 0.05 (one sided) level and statistical power to 0.9. The correlation between baseline/follow-up measurements of the outcome was set to 0.4. A sample size of *n*1 = *n*2 = *n*3 = *n*4 = *n*5 = *n*6 = 20 was estimated, to obtain a 1-point reduction in NAFLD score in the treatment groups after three months.

### 2.4. Data Collection

Trained interviewers collected information on socio-demographic aspects, medical history and lifestyle. In particular, validated questionnaires (International Physical Activity Questionnaire and European Prospective Investigation on Cancer) were used to probe alcohol intake, physical activity and eating behavior [16,17,18].

### 2.5. Randomization and Masking

**Figure 1:**
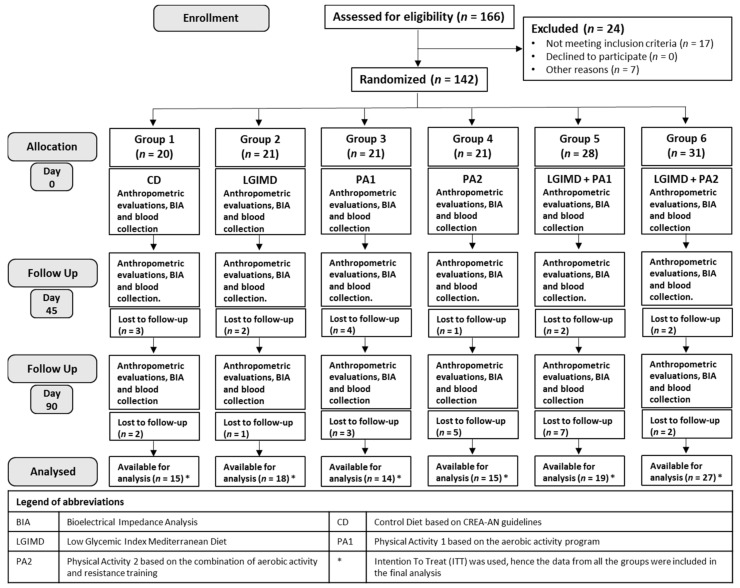
Flowchart of study design.

Participants were randomly assigned to 1 of the 6 treatment groups, according to a computerized random numbers sequence, and a one-to-one ratio was used to allocate the subjects. Blinding and equipoise were strictly maintained by emphasizing to the intervention staff and participants that each diet adhered to healthy principles. Only dietitians knew the diets assigned to subjects and although each dietitian followed the participant for the duration of the trial, individual assignment at baseline was made on a random basis. Interviewers and staff members who obtained outcome measurements, were not informed about diet or physical activity programs assigned. The 142 subjects were randomized into six groups as follows: Group 1 received a Control Diet (CD) based on CREA-AN (Research Centre for Food and Nutrition, Council for Agricultural Research and Economics, Rome, Italy) guidelines; Group 2 received a Low Glycemic Index Mediterranean Diet (LGIMD); Group 3, Physical Activity 1 aerobic program (PA1); Group 4, Physical Activity 2 combined program (aerobic activity and resistance training) (PA2); Group 5, LGIMD plus PA1 and Group 6, LGIMD plus PA2. Anthropometric evaluations, body composition measurements and blood collection were performed three times along the study on days: 0 (baseline), 45 (intermediate point) and 90 (end point). The trial flowchart is shown in Figure 1.

### 2.6. Anthropometric and Body Composition Measurements

On days 0, 45 and 90, qualified nutritionists evaluated the following anthropometric parameters: height, weight, BMI and waist circumference. All anthropometric measurements were done on subjects who wore only underwear and were standing, with joined heels, straight head, relaxed abdominal muscles, arms along the body. The weight was taken using an electronic balance SECA mod. 708 and it was approximated to the nearest 0.1 kg. The height was measured with a wall-mounted stadiometer SECA mod. 206, approximate to 1 cm. Height and weight measurements were used to calculate BMI (kg/m^2^). The waist circumference was taken using a circumference measuring tape. Bioelectrical Impedance Analysis (BIA) was used to determine body composition indirectly by measuring the impedance, given by resistance (R) and reactance (Xc), of a low-voltage current passing through the body (NUTRILAB, Akern, Firenze, Italy). Based on the European Society of Parenteral and Enteral Nutrition (ESPEN) guidelines [19], to all subjects were asked to remain in a supine position and two pairs of source and reception electrodes were placed in the standard position on the right hand and foot. The instrument applies a current of 400 μA at a constant frequency of 50 kHz. The following values were obtained from the BIA analysis: Phase Angle (PhA) calculated as arctangent Xc/R, Fat Free Mass (FFM), Fat Mass (FM), Total Body Water (TBW) and hydration (TBW/FFM).

#### 2.6.1. Blood Sample Analysis

After 12 h fasting, the participants had a blood sample taken by venous puncture at the three times (baseline, intermediate point and end point). Blood samples were collected in tubes containing Ethylenediaminetetraacetic Acid (EDTA-K2) anticoagulant for AA/EPA ratio assay, or with Silica Gel as activator of coagulation for routine analyses. All the analyses were performed within 3 months.

#### 2.6.2. AA/EPA Ratio Assay

Purified erythrocytes cell membranes were used for AA/EPA ratio assessment. Whole blood samples were stratified on a Ficoll-Paque solution and centrifuged at 400× *g* for 40 min at 20 °C. The lymphocytes and plasma were removed, while the RBC were washed with 4-volumes of phosphate-buffered saline and stored at −80 °C until their analysis. Each sample of RBC was used for fatty acid extraction and purification using the modified method of Moilanen [20], and Folch [21], as previously described [14]. Briefly, fatty acids were hydrolyzed from phospholipids of RBC membranes by adding an acidified salt solution (H_2_SO_4_ 2 × 10^–4^ M, NaCl 0.1%) and a mixture of chloroform: methanol (2:1, *v*/*v*) (Sigma-Aldrich, Milan, Italy). After centrifugation, the fatty acids contained in the lower phase were removed, transferred to a new tube and dried with a centrifugal evaporator (Bio-Rad, Milan, Italy). The Fatty Acid Methyl Esters (FAME) were obtained after derivatization with toluene and Boron trifluoride-methanol solution 14% in methanol (Sigma-Aldrich, Milan, Italy) and incubating for 2 h at 80 °C. The samples were centrifuged and the upper layer, containing FAME, was transferred into a vial and injected into a gas chromatograph (Thermo Fisher Scientific, Focus GC, Milan, Italy) equipped with auto-sampler, a split/split less injector, Flame Ionization Detector (FID) and a hydrogen gas generator (Hy Gen 200, Claind Srl, Lenno, Italy). Separation of FAME was carried out on a BPX 70 capillary column SGE Analytical Science, P/N SGE 054623, 60 m × 0.25 mm ID − BPX70 0.25 µm (SGE Europe Ltd., Milton Keynes, UK), as previously described [14]. Quantification of FAME, in particular AA and EPA methyl esters was performed using a mixture of standards (Supelco 37-Component FAME Mix, Sigma-Aldrich, Milan, Italy).

#### 2.6.3. Diet Interventions

Two types of diets were prescribed: A Control Diet (CD) based on CREA-AN guidelines [22] and a Low Glycemic Index Mediterranean Diet (LGIMD) based on consumption of bread and pasta derived from real whole meal flour (not reconstituted), vegetables and seasonal fruit, legumes, nuts, oily fish and white meats in moderate amounts, low-fat cheese and eggs weekly, and extra virgin olive oil [23]. No indications have been given regarding the total calories to be consumed, but the recommended diets were provided in brochure format, with graphical explanations organized according to a traffic light system with a list of foods that can be consumed frequently (green foods), sometimes (yellow foods) and never (red foods). All participants were obliged to record what they ate daily in a diary. The Mediterranean Adequacy Index (MAI) was chosen as a relevant measure to evaluate the adherence to both the intervention and control diets. A median value of 7.5 with an inter-quantile range (IQR) of 5.4 was, as established by the reference Italian Mediterranean Diet, expected [24]. Diet composition is shown in the Supplementary Data.

#### 2.6.4. Physical Activity Interventions

Before randomization, all subjects underwent an assessment of their level of physical activity using subjective methods, such as questionnaires and diaries; or objective methods, such as motion sensors and heart-rate monitors [25]. Furthermore, subjects performed three physical field tests [26]. Physical activity interventions included two different types of exercise programs: PA1 based on the aerobic activity program and PA2 based on the combination of aerobic activity and resistance training (Table 1). All subjects were randomized for one of the two physical activity programs based on the results of the tests performed [26]. A progressive change of the monthly target has been planned based on the results obtained.

#### 2.6.5. Measurement of NAFLD

Liver stiffness and Controlled Attenuation Parameter (CAP) measurements were performed by a vibration-controlled elastography (VCTE) implemented on FibroScan^®^ (Echosens, Paris, France) [27]. The CAP score is measured in decibels per meter (dB/m). Values < 215 dB/m corresponded to the absence of NAFLD; values between 215 and 250 dB/m indicated a mild NAFLD; values between 251 and 299 dB/m indicated a moderate NAFLD, while values ≥ 300 dB/m corresponded to a severe NAFLD [28].

### 2.7. Statistical Analysis

Intention-to-treat (ITT) principle was used to analyze and compare the intervention and control groups. As per ITT principle, for the purpose of statistical analyses, all participants retained their randomized (allocated) group, regardless of the actual treatment received. Since the assumption of normality was not satisfied for the outcome variable (AA/EPA Ratio), a logarithmic transformation was performed to ensure that the assumption is satisfied. All data were expressed as mean (±SD) or percentages for descriptive purpose. ANOVA and Chi-squared test were used to test differences between continuous and categorical variables, respectively. ANOVA and Dunnett’s post hoc test were used where appropriate. Linear mixed-effects models were performed to estimate the association between different variables and the outcome (AA/EPA Ratio). The results obtained are expressed in natural scale as β coefficient and 95% CI (Confidence Interval). The post-estimation tool—*margins*—was then used to estimate the predicted value of AA/EPA Ratio by intervention group and time. Statistical analysis was performed by using Stata statistical software, version 15.1 (StataCorp, 4905 Lakeway Drive, College Station, TX 77845, USA).

## 3. Results

One hundred forty-two subjects, 57 females and 85 males, were randomized to six study groups and their characteristics at baseline are summarized in Table 2 and Table 3. Mean age was 51.3 (9.2) all subjects were overweight/obese with high Waist Circumference. CAP was over 297 dB/m in all groups with low levels of HDL (High-Density Lipoprotein) and high HOMA-IR (Homeostatic Model Assessment for Insulin Resistance). Most subjects were male, former or current smokers, married or divorced with secondary school or higher. For all parameters considered, at the baseline no differences were detected among groups, indicating effective randomization. A flowchart illustrating the study design of patients is shown in Figure 1. Adherence to physical activity programs is shown in Table 4. There was an important adherence to both aerobic and combined physical activity programs. Results from field fitness test by age class, sex and time are shown in Table 5. Overall there was a statistically significant improvement in all age classes by both men and women with the exception of the Sit and Reach test.

**Figure 2:**
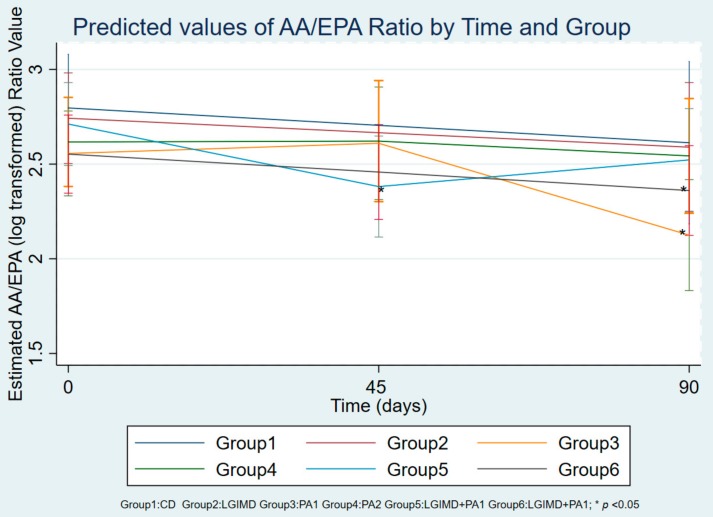
Predictive margins of time versus groups with 95% Confidence Intervals. Estimated logarithmic value of the AA/EPA ratio in the 6 groups at day 0 (baseline), day 45 (intermediate point) and day 90 (end point). Group 1 received only a Control Diet (CD) based on CREA-AN guidelines; Group 2 received only a Low Glycemic Index Mediterranean Diet (LGIMD); Group 3 was subjected to Physical Activity 1 (PA1) based on the aerobic activity program without following a specific diet; Group 4 was subjected to Physical Activity 2 (PA2) based on the combination of aerobic activity and resistance training without following a specific diet; Group 5 followed a LGIMD combined with a PA1 program; Group 6 followed a LGIMD combined with a PA2 program.

Table 6 shows the changes in the levels of main fatty acids studied at baseline, intermediate point and end point in the treatment groups. Our outcome was the AA/EPA ratio. In order to evaluate any association between AA/EPA ratio value and each of the metabolic variables considered, a multiple linear regression model was performed (Table 7). A statistically significant reduction of AA/EPA ratio was observed in Group 3 after 90 days of treatment with only PA1 (Table 7). Moreover, a significant decrease of AA/EPA ratio was present in the subjects enrolled in Group 6 at both 45 and 90 days (Table 7). Interestingly, different behavior of the AA/EPA ratio value was detected in Group 5, where the combination of PA1 program with LGIMD reduced the ratio value after only 45 days of treatment (Table 7). In the final linear regression model, a significance was observed for BMI and CAP values, demonstrating that the reduction of AA/EPA ratio levels was associated with a lower BMI and an improvement of NAFLD. Table 8 and Figure 2 show the predictive margins of AA/EPA ratio values over time (days 0, 45 and 90) for the 6 groups with 95% Confidence Intervals. Group 3 shows a marked reduction in the AA/EPA ratio after 90 days of treatment with only PA1. In Group 5, the treatment with LGIMD associated with PA1 program exerted a significant reduction of AA/EPA ratio after only 45 days, however, no effect was present after 90 days. Moreover, compared to the baseline values, a slow decrease of AA/EPA ratio was present in the subjects enrolled in the Group 6, demonstrating that the combined effect of LGIMD and PA2, based on the aerobic activity and resistance training, caused a slow, but constant reduction over time.

## 4. Discussion

To our best knowledge, this is the first study to investigate the AA/EPA ratio value in erythrocyte membranes of subjects enrolled in a nutritional clinical trial associated with two different programs of physical activity. Although the mechanism underlying the association between AA/EPA ratio and NAFLD has not been clearly defined, our data suggest that the inflammatory effects of AA contributes to the pathogenesis of liver injury. These findings imply that the intake of EPA-rich foods could be effective for reducing the inflammatory insult due to high levels of AA in cell membranes. Furthermore, there is evidence regarding the role of omega-6 and omega-3 PUFAs in regulating cell proliferation. In a recent study conducted on human breast adenocarcinoma cell lines (MCF7 and MDA-MB-231), a low AA/EPA ratio has been shown to modulate cancer growth by increasing the expression of tumor suppressors [29]. Moreover, different studies have shown a direct relationship between AA levels and chronic inflammation, a condition that occurs in obesity and liver disease [9]. As opposed, EPA has been demonstrated to prevent obesity increasing thermogenesis through uncoupling protein 1 (UCP-1), which represents a biomarker for brown adipose tissue (BAT) [30]. Previously, we observed a lower percentage of total omega-3 PUFAs in RBC membranes of patients with colorectal cancer (CRC). Compared to subjects with no malignant disease, CRC patients showed an altered fatty acid profile, particularly in a lower omega-3/omega-6 PUFAs ratio [31].

In this study, we observed that the balance between AA and EPA in cell membranes is also regulated by physical activity, in accord with other studies demonstrating that an increase in physical activity improves metabolic comorbidities, including cardiovascular disease, diabetes, metabolic syndrome and dyslipidemia [32,33]. Moreover, the physical activity programs were also able to improve the score of steatosis reducing the CAP value assessed by Fibroscan. Exercise might interfere with the synthesis of fatty acids, exerting a beneficial effect on liver metabolism.

In particular, it appears that the combination of PA1 and LGIMD (Group 5) was initially more efficacious in reducing AA/EPA levels at 45 days of treatment. However, over the full 90 days, a consistent, gradual reduction over time of AA/EPA ratio was present in the subjects enrolled in Group 6, demonstrating that the combined effect of LGIMD and PA2, based on aerobic activity and resistance training, has beneficial effects on the inflammatory state. The mechanism proposed for this synergistic effect is the reduction of plasma lipids, improving hepatic injury, fitness and body composition. It is possible that omega-3 PUFAs present in large amounts in LGIMD, were able to exert an anti-inflammatory effect on cell membranes, causing a general wellbeing in the subjects.

Some methodological issues need to be considered. Sample size, randomization of intervention and measured adherence to interventions constitute strengths of this study. Unfortunately, we have not measured all other inflammation biomarkers involved in the pathogenesis of NAFLD. However, the distribution of these factors should be equally distributed among groups because of randomization.

## 5. Conclusions

Since inflammation is at the base of many chronic diseases, including NAFLD, dietary intake of omega-3 PUFAs associated with a healthy lifestyle, could be used in prevention protocols for obesity, cardiovascular diseases, as well as liver diseases and cancer. Moreover, the analysis of fatty acid profiles in cell membranes, which is an easy to perform method, may contribute to better characterize at risk subjects and the cellular states.

## Figures and Tables

**Table 1:** Description of exercise interventions. All subjects were randomized for one of the two physical activity programs based on the results of the tests performed: PA1 based on the aerobic activity program and PA2 based on the combination of aerobic activity and resistance training. The total weekly exercise duration was expressed in minutes (min).

	Physical Activity Groups	
	PA1	PA2
*N*. of weekly sessions	4 ^a^	3 ^b^
Type of Exercise: Aerobic	45–60 min of moderate-intensity treadmill walking or cycling (60–75% VO_2_ max obtained from HRM).	45 min of moderate-intensity treadmill walking or cycling (60–75% VO_2_ max obtained from HRM).
Type of Exercise: Resistance		2 sets of 12 exercises, each to volitional fatigue: leg press; adductor/abductor; buttocks; bicep curls, tricep extensions, three different abdominal exercises, leg machine, low row, shoulder flexion.Approximate duration of each session 45 min.
Total Weekly Exercise	180–240 min	270 min

^a^ Sessions were performed on nonconsecutive days of the week. ^b^ The weight lifting was increased when 10–12 repetitions were completed with good form. Abbreviations: PA1, Physical Activity 1; PA2, Physical Activity 2; HRM, Heart Rate Monitor; VO_2_, Oxygen consumption per unit of time.

**Table 2:** Descriptive statistics (mean ± Standard Deviation) of the main characteristics of the subjects with NAFLD at baseline, randomized in six study groups: Group 1 received only a CD based on CREA-AN guidelines; Group 2 received only a LGIMD; Group 3 was subjected to PA1 based on the aerobic activity program without following a specific diet; Group 4 was subjected to PA2 based on the combination of aerobic activity and resistance training without following a specific diet; Group 5 followed a LGIMD combined with a PA1 program; Group 6 followed a LGIMD combined with a PA2 program (continuous variables).

Variables *	Group 1 CD	Group 2 LGIMD	Group 3 PA1	Group 4 PA2	Group 5 LGIMD + PA1	Group 6 LGIMD + PA2
AA/EPA, %	18.5 ± 10.1	18.2 ± 8.11	16.3 ± 7.01	16.4 ± 6.79	18.0 ± 8.87	14.6 ± 4.52
Age, years	52.1 ± 9.52	55.5 ± 10.4	50.8 ± 9.62	45.9 ± 9.09	49.5 ± 9.27	46.3 ± 11.1
PhA, degrees	6.06 ± 0.89	6.16 ± 1.01	6.19 ± 0.85	6.44 ± 0.73	6.12 ± 0.90	6.11 ± 0.87
FM, kg	34.2 ± 12.2	30.9 ± 10.4	31.1 ± 10.5	28.1 ± 7.13	31.0 ± 7.64	37.0 ± 12.1
Hydration, %	73.9 ± 1.76	73.9 ± 2.32	73.6 ± 1.23	73.4 ± 0.48	73.8 ± 1.45	74.2 ± 2.10
BMI, kg/m^2^	34.1 ± 4.97	32.8 ± 4.22	32.7 ± 5.24	30.8 ± 3.16	33.1 ± 4.20	35.4 ± 5.34
Waist Circumference, cm	105.9 ± 11.9	102.1 ± 11.4	104.1 ± 8.49	99.7 ± 8.10	103.5 ± 11.9	106.4 ± 13.7
Cholesterol, mg/dL	199.1 ± 40.8	199.1 ± 53.8	193.1 ± 31.1	205.4 ± 35.9	202.9 ± 42.5	199.4 ± 38.6
CAP, dB/m	297.9 ± 77.2	324.9 ± 42.5	313.0 ± 46.5	316.4 ± 41.5	307.8 ± 38.7	303.0 ± 36.4
HDL, mg/dL	47.5 ± 13.1	47.1 ± 15.1	43.4 ± 10.2	44.9 ± 11.6	44.1 ± 8.38	43.2 ± 10.5
Triglycerides, mg/dL	106.5 ± 53.4	142.1 ± 104.4	125.0 ± 86.3	123.1 ± 58.3	144.6 ± 93.5	141.0 ± 82.4
HOMA-IR, mg/dL	2.90 ± 1.65	2.74 ± 1.29	2.95 ± 1.67	2.51 ± 1.59	3.23 ± 1.76	3.59 ± 2.70
Hemoglobin, g/L	14.5 ± 1.42	14.6 ± 1.11	14.7 ± 1.00	15.0 ± 1.16	14.4 ± 1.57	14.3 ± 1.59
Hematocrit, %	42.8 ± 3.84	43.4 ± 2.64	42.9 ± 2.41	43.7 ± 3.18	42.6 ± 3.48	42.3 ± 3.77
Neutrophils, 10^3^/µL	3.26 ± 0.81	3.94 ± 1.65	3.87 ± 1.39	3.47 ± 1.30	3.87 ± 1.11	4.05 ± 1.31
SFAs, %	54.2 ± 9.10	51.83 ± 6.79	52.46 ± 8.67	53.55 ± 10.94	52.40 ± 8.21	51.86 ± 7.10
MUFAs, %	21.25 ± 7.41	21.1 ± 3.96	22.32 ± 6.08	21.79 ± 5.47	23.26 ± 5.46	22.70 ± 4.82
PUFAs, %	24.5 ± 4.90	26.1 ± 5.49	24.6 ± 5.45	24.9 ± 5.89	24.2 ± 5.23	24.6 ± 4.85
Palmitic acid, %	29.37 ± 5.16	28.28 ± 3.04	28.26 ± 5.13	28.06 ± 4.31	27.99 ± 3.93	27.13 ± 4.69
Stearic acid, %	20.09 ± 4.60	18.78 ± 4.34	18.75 ± 4.29	20.11 ± 8.17	20.16 ± 5.64	18.39 ± 3.36
Oleic acid, %	15.79 ± 6.86	13.18 ± 2.76	15.67 ± 4.79	14.64 ± 5.06	14.28 ± 4.74	15.88 ± 3.70
DGLA, %	1.34 ± 0.36	1.55 ± 0.47	1.40 ± 0.26	1.31 ± 0.52	1.43 ± 0.32	1.50 ± 0.63
DHA, %	2.44 ± 0.67	2.99 ± 0.88	3.07 ± 0.78	2.51 ± 0.79	2.75 ± 0.92	3.04 ± 0.55
AA, %	12.6 ± 6.38	13.16 ± 6.99	10.9 ± 4.97	10.5 ± 4.31	11.7 ± 3.94	11.7 ± 3.77
EPA, %	0.72 ± 0.21	0.74 ± 0.21	0.72 ± 0.26	0.70 ± 0.22	0.72 ± 0.25	0.85 ± 0.26

* ANOVA Test. *p* > 0.10 for all Variables by Intervention Group. Abbreviations: CD, Control Diet; LGIMD, Low Glycemic Index Mediterranean Diet; PA1, Physical Activity 1; PA2, Physical Activity 2; PhA, Phase Angle; FM, Fat Mass; BMI, Body Mass Index; CAP, Controlled Attenuation Parameter (marker of steatosis); HDL, High-Density Lipoprotein; HOMA-IR, Homeostatic Model Assessment for Insulin Resistance; SFAs, Saturated Fatty Acids; MUFAs, Monounsaturated fatty acids; PUFAs, Polyunsaturated fatty acids; DGLA, Dihomo-gamma-linolenic acid; DHA, Docosahexaenoic acid; AA, Arachidonic acid; EPA, Eicosapentaenoic acid.

**Table 3:** Descriptive statistics (mean ± standard deviation) of the main characteristics of the subjects with NAFLD at baseline, randomized in six study groups: Group 1 received only a CD based on CREA-AN guidelines; Group 2 received only a LGIMD; Group 3 was subjected to PA1 based on the aerobic activity program without following a specific diet; Group 4 was subjected to PA2 based on the combination of aerobic activity and resistance training without following a specific diet; Group 5 followed a LGIMD combined with a PA1 program; Group 6 followed a LGIMD combined with a PA2 program (categorical variables)*.*

Variables *	Group 1 % (SD%)		Group 2 % (SD%)		Group 3 % (SD%)		Group 4 % (SD%)		Group 5 % (SD%)		Group 6 % (SD%)	
**Gender**												
Female	10	(17.9)	8	(12.5)	8	(16.1)	8	(10.7)	8	(16.1)	15	(26.8)
Male	10	(10.7)	13	(15.5)	13	(16.7)	13	(16.7)	20	(19.0)	16	(21.4)
**Smoking status**												
Never smoked	12	(16.4)	7	(9.6)	10	(13.7)	10	(13.7)	16	(21.9)	18	(24.7)
Former smoker	4	(12.9)	7	(22.6)	5	(16.1)	4	(12.9)	4	(12.9)	7	(22.6)
Current smoker	0	(0.0)	4	(17.4)	5	(21.7)	4	(17.4)	3	(13.0)	7	(30.4)
**Level of Physical activity**												
Low	0	(0.0)	0	(0.0)	1	(11.1)	3	(33.3)	1	(11.1)	4	(44.4)
Moderate	4	(13.8)	5	(17.2)	3	(10.3)	4	(13.8)	4	(13.8)	9	(31.0)
High	12	(14.1)	13	(15.3)	14	(16.5)	11	(12.9)	17	(20.0)	18	(21.2)
**Status**												
Single	1	(7.7)	1	(7.7)	1	(7.7)	3	(23.1)	2	(15.4)	5	(38.5)
Married	14	(14.6)	15	(15.6)	17	(17.7)	11	(11.5)	17	(17.7)	22	(22.9)
Divorced	0	(0.0)	0	(0.0)	1	(25.0)	0	(0.0)	1	(25.0)	2	(50.0)
Widowed	0	(0.0)	0	(0.0)	0	(0.0)	2	(100.0)	0	(0.0)	0	(0.0)
**Study level**												
Elementary	1	(25.0)	1	(25.0)	0	(0.0)	0	(0.0)	1	(25.0)	1	(25.0)
Secondary School	8	(21.6)	6	(16.2)	8	(21.6)	3	(8.1)	4	(10.8)	8	(21.6)
High School	5	(9.3)	8	(14.8)	8	(14.8)	9	(16.7)	7	(13.0)	17	(31.5)
Diploma college	1	(50.0)	1	(50.0)	0	(0.0)	0	(0.0)	0	(0.0)	0	(0.0)
University degree	0	(0.0)	1	(4.8)	3	(14.3)	5	(23.8)	8	(38.1)	4	(19.0)
**NAFLD**												
Moderate	5	(11.1)	5	(11.1)	6	(13.3)	7	(15.6)	9	(20.0)	13	(28.9)
Severe	14	(14.7)	15	(15.8)	17	(17.9)	13	(13.7)	16	(16.8)	20	(21.1)
**Diabetes**												
No	16	(14.3)	15	(13.4)	21	(18.8)	18	(16.1)	15	(13.4)	27	(24.1)
Yes	1	(11.1)	3	(33.3)	1	(11.1)	0	(0.0)	3	(33.3)	1	(11.1)
**Hypertension**												
No	10	(12.7)	9	(11.4)	14	(17.7)	13	(16.5)	12	(15.2)	21	(26.6)
Yes	7	(15.9)	9	(20.5)	8	(18.2)	5	(11.4)	8	(18.2)	7	(15.9)
**Cancer**												
No	14	(12.4)	16	(14.2)	19	(16.8)	18	(15.9)	20	(17.7)	26	(23.0)
Yes	3	(37.5)	1	(12.5)	2	(25.0)	0	(0.0)	0	(0.0)	2	(25.0)

* Chi-squared Test: *p* > 0.10 for all Variables by Intervention Group. Abbreviation: NAFLD, Non-Alcoholic Fatty Liver Disease. Some cells do not add up to the total number because of missing data.

**Table 4:** Adherence to physical activity programs by intervention group, sex and week from enrollment.

Week		Group 3		Group 4		Group 5		Group 6	
		Male	Female	Male	Female	Male	Female	Male	Female
		% (SE%)	% (SE%)	% (SE%)	% (SE%)	% (SE%)	% (SE%)	% (SE%)	% (SE%)
3rd	Time	91.4 ± 17.1	100 ± 0.00	100 ± 0.00	100 ± 0.00	98.1 ± 25.9	99.9 ± 23.5	100 ± 0.00	100 ± 0.00
	Intensity	99.3 ± 3.72	100 ± 0.00	100 ± 0.00	100 ± 0.00	100 ± 0.00	100 ± 0.00	100 ± 0.00	100 ± 0.00
	Load	N/A	N/A	100 ± 0.00	100 ± 0.00	N/A	N/A	100 ± 0.00	100 ± 0.00
4th	Time	98.0 ± 40.1	125 ± 41.8	100 ± 0.00	100 ± 0.00	92.0 ± 15.6	86.3 ± 16.5	100 ± 0.00	100 ± 0.00
	Intensity	99.0 ± 3.33	100 ± 0.00	100 ± 0.00	100 ± 0.00	100 ± 0.00	100 ± 0.00	100 ± 0.00	100 ± 0.00
	Load	N/A	N/A	100 ± 0.00	100 ± 0.00	N/A	N/A	100 ± 0.00	100 ± 0.00
5th	Time	88.4 ± 15.7	97.3 ± 31.0	104.2 ± 14.4	108.3 ± 20.4	90.0 ± 18.5	91.0 ± 17.2	100 ± 0.00	100 ± 0.00
	Intensity	99.3 ± 2.13	100 ± 0.00	100 ± 0.00	100 ± 0.00	100 ± 0.00	100 ± 0.00	100 ± 0.00	100 ± 0.00
	Load	N/A	N/A	100 ± 0.00	100 ± 0.00	N/A	N/A	100 ± 0.00	100 ± 0.00
6th	Time	96.1 ± 20.4	90.0 ± 16.4	104.2 ± 14.4	108.3 ± 20.4	87.0 ± 19.0	93.0 ± 14.0	100 ± 0.00	100 ± 0.00
	Intensity	99.9 ± 3.19	100 ± 0.00	100 ± 0.00	100 ± 0.00	100 ± 0.00	100 ± 0.00	100 ± 0.00	100 ± 0.00
	Load	N/A	N/A	100 ± 0.00	100 ± 0.00	N/A	N/A	100 ± 0.00	100 ± 0.00
7th	Time	89.0 ± 19.2	129.5 ± 72.2	108.3 ± 19.5	108.3 ± 20.4	93.1 ± 29.1	116.0 ± 69.5	100 ± 0.00	100 ± 0.00
	Intensity	99.7 ± 0.83	100 ± 0.00	100 ± 0.00	100 ± 0.00	100 ± 0.00	100 ± 0.00	100 ± 0.00	100 ± 0.00
	Load	N/A	N/A	100 ± 0.00	100 ± 0.00	N/A	N/A	100 ± 0.00	100 ± 0.00
8th	Time	112.2 ± 47.2	112.5 ± 30.6	100 ± 0.00	100 ± 0.00	80.0 ± 21.9	121.1 ± 77.3	100 ± 0.00	100 ± 0.00
	Intensity	99.9 ± 1.07	100 ± 0.00	100 ± 0.00	100 ± 0.00	100 ± 0.00	100 ± 0.00	100 ± 0.00	100 ± 0.00
	Load	N/A	N/A	100 ± 0.00	100 ± 0.00	N/A	N/A	100 ± 0.00	100 ± 0.00
9th	Time	103.4 ± 39.5	110.0 ± 20.0	109.1 ± 20.2	112.5 ± 25.0	88.3 ± 36.0	102.2 ± 33.5	103.5 ± 13.4	100 ± 0.00
	Intensity	100.5 ± 1.39	100 ± 0.00	100 ± 0.00	100 ± 0.00	100 ± 0.00	100 ± 0.00	100 ± 0.00	100 ± 0.00
	Load	N/A	N/A	100 ± 0.00	100 ± 0.00	N/A	N/A	100 ± 0.00	100 ± 0.00
10th	Time	93.4 ± 15.5	134.3 ± 32.5	109.1 ± 20.2	112.5 ± 25.0	88.3 ± 36.0	96.1 ± 27.5	103.6 ± 13.4	100 ± 0.00
	Intensity	100.5 ± 1.39	100 ± 0.00	100 ± 0.00	100 ± 0.00	100 ± 0.00	100 ± 0.00	100 ± 0.00	100 ± 0.00
	Load	N/A	N/A	100 ± 0.00	100 ± 0.00	N/A	N/A	100 ± 0.00	100 ± 0.00

Group 3 was subjected to PA1 based on the aerobic activity program; Group 4 was subjected to PA2 based on the combination of aerobic activity and resistance training; Group 5 followed a LGIMD combined with a PA1 program; Group 6 followed a LGIMD combined with a PA2 program. N/A: not applicable.

**Table 5:** Field fitness results by age class, sex and time.

Age Classes (Years)	Test		Men	Women	
		Baseline	3rd Month	Baseline	3rd Month
		Mean ± SD	Mean ± SD	Mean ± SD	Mean ± SD
30–39	Walking (s) *	1105.5 ± 76.1	1020.5 ± 64.2	1283.7 ± 116.3	1150.0 ± 142.0
	Push-up *	12.1 ± 3.89	18.9 ± 4.73	9.67 ± 10.6	26.6 ± 10.8
	Sit and Reach	−9.41 ± 7.24	−6.87 ± 6.22	−5.83 ± 4.87	−2.60 ± 6.11
40–49	Walking (s) *	1102.2 ± 96.9	1033.7 ± 94.4	1264.6 ± 211.8	1115.4 ± 124.5
	Push-up *	10.8 ± 7.37	15.7 ± 7.47	10.8 ± 8.96	16.3 ± 9.76
	Sit and Reach	−11.9 ± 9.64	−7.25 ± 11.5	−6.20 ± 9.18	−6.82 ± 10.4
50–59	Walking (s) *	1106.6 ± 167.6	1035.8 ± 81.8	1367.7 ± 165.7	1195.4 ± 66.5
	Push-up *	10.3 ± 7.39	17.3 ± 8.72	6.54 ± 5.54	18.8 ± 7.93
	Sit and Reach	−9.55 ± 6.78	−6.12 ± 8.70	−9.54 ± 10.7	−6.22 ± 8.84
≥60	Walking (s) *	1173.2 ± 102.4	1103.5 ± 84.5	1311.3 ± 78.6	1235.4 ± 96.5
	Push-up *	5.29 ± 3.80	10.00 ± 7.65	5.83 ± 5.25	11.8 ± 4.52
	Sit and Reach	−14.1 ± 9.53	−14.7 ± 7.28	−7.75 ±7.87	−6.67 ± 7.52

* Kruskal-Wallis equality-of-populations rank test: *p* < 0.05. SD: Standard Deviation.

**Table 6:** Mean percentage of main fatty acids in red blood cell membranes of the subjects with NAFLD at baseline (day 0), intermediate point (day 45) and end point (day 90), randomized in six treatment groups.

Fatty Acids	Time	Group 1 CD	Group 2 LGIMD	Group 3 PA1	Group 4 PA2	Group 5 LGIMD + PA1	Group 6 LGIMD + PA2
AA/EPA, %	Day 0	18.5 ± 10.1	18.2 ± 8.11	16.3 ± 7.01	16.4 ± 6.79	18.0 ± 8.87	14.6 ± 4.52
	Day 45	14.4 ± 5.34	14.7 ± 8.14	13.0 ± 7.08	13.5 ± 5.95	10.4 ± 5.10 *	12.8 ± 7.00
	Days 90	17.8 ± 6.60	17.2 ± 8.46	11.3 ± 6.90 *	15.9 ± 7.20	12.5 ± 4.80 *	11.1 ± 4.70 *
AA, %	Day 0	12.6 ± 6.38	13.1 ± 6.99	10.9 ± 4.97	10.5 ± 4.31	11.7 ± 3.94	11.7 ± 3.77
	Day 45	13.8 ± 1.90	12.9 ± 3.35	13.1 ± 3.52	12.8 ± 3.68	14.0 ± 2.80 *	10.9 ± 3.50
	Days 90	13.3 ± 3.16	12.4 ± 3.28	12.4 ± 3.20	13.8 ± 3.60 *	13.8 ± 2.60 *	10.3 ± 2.70
EPA, %	Day 0	0.7 ± 0.21	0.7 ± 0.21	0.7 ± 0.26	0.7 ± 0.22	0.7 ± 0.25	0.8 ± 0.26
	Day 45	1.1 ± 0.44	1.1 ± 0.57 *	1.2 ± 0.68 *	1.1 ± 0.47 *	1.5 ± 0.50 *	1.3 ± 1.30
	Days 90	0.9 ± 0.76	0.8 ± 0.41	1.5 ± 0.98 *	1.0 ± 0.40 *	1.2 ± 0.40 *	1.2 ± 0.90
Palmitic acid, %	Day 0	29.3 ± 5.16	28.2 ± 3.04	28.2 ± 5.13	28.0 ± 4.31	27.9 ± 3.93	27.1 ± 4.69
	Day 45	22.9 ± 1.53 *	25.4 ± 3.17 *	23.5 ± 1.97	25.5 ± 3.24	25.6 ± 4.17	22.1 ± 2.22 *
	Days 90	27.5 ± 1.69	29.0 ± 2.77	26.4 ± 3.52	27.1 ± 3.73	26.2 ± 3.44	26.0 ± 4.13
Stearic acid, %	Day 0	20.1 ± 4.60	18.7 ± 4.34	18.7 ± 4.29	20.1 ± 8.17	20.1 ± 5.64	18.3 ± 3.36
	Day 45	15.5 ± 4.56 *	16.9 ± 4.13	17.8 ± 2.25	18.8 ± 5.60	18.6 ± 5.83	16.3 ± 2.36 *
	Days 90	16.6 ± 1.19 *	18.9 ± 4.17	16.3 ± 1.28	19.4 ± 5.88	18.4 ± 4.75	18.2 ± 2.48
Oleic acid, %	Day 0	15.8 ± 6.86	13.1 ± 2.76	15.6 ± 4.79	14.6 ± 5.06	14.2 ± 4.74	15.8 ± 3.70
	Day 45	15.6 ± 4.47	16.2 ± 3.46 *	14.9 ± 2.59	14.5 ± 3.81	13.9 ± 3.00	16.1 ± 3.22
	Days 90	15.8 ± 3.18	13.5 ± 2.34	15.3 ± 3.39	13.9 ± 3.13	14.5 ± 4.37	16.6 ± 3.49
DGLA, %	Day 0	1.3 ± 0.36	1.5 ± 0.47	1.4 ± 0.26	1.3 ± 0.52	1.4 ± 0.32	1.5 ± 0.63
	Day 45	2.0 ± 0.95 *	1.1 ± 0.32	1.9 ± 0.54 *	1.2 ± 0.40	1.4 ± 0.62	1.6 ± 0.73
	Days 90	1.5 ± 0.59	1.3 ± 0.42	1.3 ± 0.31	1.5 ± 0.60	1.5 ± 0.68	1.5 ± 0.46
DHA, %	Day 0	2.4 ± 0.67	2.9 ± 0.88	3.0 ± 0.78	2.5 ± 0.79	2.7 ± 0.92	3.0 ± 0.55
	Day 45	3.9 ± 0.47 *	3.5 ± 0.71 *	3.7 ± 1.04 *	3.4 ± 0.54 *	3.9 ± 1.27 *	4.1 ± 1.00 *
	Days 90	3.0 ± 0.60 *	3.0 ± 0.68	3.5 ± 0.84	3.0 ± 0.88	3.5 ± 1.34	3.5 ± 0.93 *
SFAs, %	Day 0	54.2 ± 9.10	51.8 ± 6.79	52.4 ± 8.67	53.5 ± 10.94	52.4 ± 8.21	51.8 ± 7.10
	Day 45	44.2 ± 4.55 *	47.6 ± 5.95 *	47.5 ± 3.27 *	50.1 ± 7.42	49.5 ± 8.78	44.8 ± 3.91 *
	Days 90	49.4 ± 2.93 *	52.6 ± 6.01	49.2 ± 5.14	52.3 ± 7.90	49.6 ± 6.80	49.4 ± 4.60
MUFAs, %	Day 0	21.2 ± 7.41	21.1 ± 3.96	22.3 ± 6.08	21.7 ± 5.47	23.2 ± 5.46	22.7 ± 4.82
	Day 45	26.2 ± 5.28 *	25.2 ± 4.75	26.3 ± 3.58	23.9 ± 4.80	24.4 ± 4.33	27.6 ± 3.72 *
	Days 90	22.5 ± 3.18	20.2 ± 2.69	23.9 ± 4.98	21.9 ± 4.73	24.6 ± 5.35	24.7 ± 5.34
PUFAs, %	Day 0	24.5 ± 4.90	26.1 ± 5.49	24.6 ± 5.45	24.9 ± 5.89	24.2 ± 5.23	24.6 ± 4.85
	Day 45	29.4 ± 2.35 *	27.0 ± 2.68	26.2 ± 2 .73	25.9 ± 3.93	26.0 ± 5.18	27.6 ± 3.72 *
	Days 90	28.0 ±1.91 *	27.0 ± 4.03	27.1 ± 2.00	25.7 ± 5.33	25.7 ± 3.67	25.8 ± 2.90

* ANOVA and Dunnett’s test: *p* < 0.05. All values are expressed as mean ± Standard Deviation. Abbreviations: CD, Control Diet; LGIMD, Low Glycemic Index Mediterranean Diet; PA1, Physical Activity 1; PA2, Physical Activity 2; AA, Arachidonic acid; EPA, Eicosapentaenoic acid; DGLA, Dihomo-gamma-linolenic acid; DHA, Docosahexaenoic acid; SFAs, Saturated Fatty Acids; MUFAs, Monounsaturated fatty acids; PUFAs, Polyunsaturated fatty acids.

**Table 7:** Multiple Mixed-Linear Regression models of AA/EPA ratio at 45 days and 90 days of treatment, controlling for age and gender.

AA/EPA Ratio (log)	β	SE	*p* Value	(95% Conf. Interval)	
AA/EPA ratio Group 5 _45 days_	−0.45	0.20	0.033	−0.83	−0.06
AA/EPA ratio Group 6 _45 days_	−0.32	0.19	0.090	−0.70	0.05
AA/EPA ratio Group 3 _90 days_	−0.67	0.20	0.001	−1.06	−0.28
AA/EPA ratio Group 6 _90 days_	−0.44	0.19	0.019	−0.81	−0.07
Triglycerides, mg/dL	0.001	0.000	0.05	0.00	0.002
Cholesterol, mg/dL	0.000	0.001	0.681	−0.003	0.002
HOMA-IR, mg/dL	0.004	0.013	0.744	−0.02	0.03
HDL, mg/dL	0.000	0.004	0.953	−0.01	0.01
Hemoglobin, g/L	0.011	0.051	0.028	0.01	0.22
Hematocrit, %	−0.06	0.032	0.041	−0.12	−0.002
Neutrophils, 10^3^/µL	0.08	0.035	0.031	0.01	0.14
CAP, dB/m	0.002	0.00	0.05	0.001	0.003
BMI, kg/m^2^	0.04	0.019	0.033	−0.08	−0.003
FM, kg	0.019	0.010	0.072	0.00	0.04
Waist Circumference, cm	0.000	0.007	0.988	−0.01	0.01

AA/EPA ratio Group 5 _45 days_: value of AA/EPA ratio in Group 5 who followed a LGIMD combined with a PA1 program at 45 days. AA/EPA ratio Group 6 _45 days_: value of AA/EPA ratio in Group 6 who followed a LGIMD combined with a PA2 program at 45 days. AA/EPA ratio Group 3 _90 days_: value of AA/EPA ratio in Group 3 who followed a PA1 program at 90 days. AA/EPA ratio Group 6 _90 days_: value of AA/EPA ratio in Group 6 who followed a LGIMD combined with a PA2 program at 90 days. Abbreviations: SE(β), standard error of coefficient; HOMA-IR, Homeostatic Model Assessment for Insulin Resistance; HDL, High-Density Lipoprotein; CAP, Controlled Attenuation Parameter (marker of steatosis); BMI, Body Mass Index; FM, Fat Mass; LGIMD, Low Glycemic Index Mediterranean Diet; PA1, Physical Activity 1; PA2, Physical Activity 2.

**Table 8:** Predictive margins of time versus groups with 95% Confidence Intervals.

Time # Group *	Margin	SE	*p*-Value	(95% Conf. Interval)	
1 # 1	2.80	0.14	0.00	2.53	3.08
1 # 2	2.74	0.12	0.00	2.51	2.97
1 # 3	2.53	0.11	0.00	2.31	2.75
1 # 4	2.65	0.12	0.00	2.42	2.89
1 # 5	2.70	0.11	0.00	2.48	2.91
1 # 6	2.53	0.10	0.00	2.33	2.73
2 # 1	Not estimable				
2 # 2	Not estimable				
2 # 3	2.63	0.15	0.00	2.34	2.92
2 # 4	2.62	0.16	0.00	2.30	2.93
2 # 5	2.35	0.13	0.00	2.09	2.62
2 # 6	2.48	0.13	0.00	2.23	2.72
3 # 1	2.63	0.22	0.00	2.20	3.05
3 # 2	2.58	0.17	0.00	2.24	2.91
3 # 3	2.13	0.15	0.00	1.83	2.42
3 # 4	2.54	0.15	0.00	2.24	2.84
3 # 5	2.52	0.13	0.00	2.26	2.78
3 # 6	2.36	0.12	0.00	2.13	2.59

* Estimated logarithmic value of the AA/EPA ratio in the 6 groups at day 0 (baseline), day 45 (intermediate point) and day 90 (end point). # *versus.* Group 1 received only a Control Diet (CD) based on CREA-AN guidelines; Group 2 received only a Low Glycemic Index Mediterranean Diet (LGIMD); Group 3 was subjected to Physical Activity 1 (PA1) based on the aerobic activity program without following a specific diet; Group 4 was subjected to Physical Activity 2 (PA2) based on the combination of aerobic activity and resistance training without following a specific diet; Group 5 followed a LGIMD combined with a PA1 program; Group 6 followed a LGIMD combined with a PA2 program.
